# Depression among Black Youth; Interaction of Class and Place

**DOI:** 10.3390/brainsci8060108

**Published:** 2018-06-12

**Authors:** Shervin Assari, Frederick X. Gibbons, Ronald Simons

**Affiliations:** 1 Department of Psychology, University of California, Los Angeles (UCLA), Los Angeles, CA 90095, USA; 2 BRITE Center for Science, Research and Policy, University of California, Los Angeles (UCLA), Los Angeles, CA 90095, USA; 3 Department of Psychiatry, University of Michigan, Ann Arbor, MI 48109, USA; 4 Center for Research on Ethnicity, Culture and Health, School of Public Health, University of Michigan, Ann Arbor, MI 48109, USA; 5 Department of Psychological Sciences, University of Connecticut, Storrs, CT 06269, USA; rick.gibbons@uconn.edu; 6 Department of Sociology, University of Georgia, Athens, GA 30602, USA; rsimons@uga.edu

**Keywords:** race, Blacks, African Americans, social class, socioeconomic status (SES), income, place, depression

## Abstract

Although high socioeconomic status (SES) is traditionally conceptualized as a health protective factor, recent literature has documented positive associations between SES (e.g., income) and depression among Blacks, including Black youth. To extend the results of this recent literature, the current study used the Family and Community Health Study (FACHS) data to examine the multiplicative effects of gender, place, and SES on average depressive symptoms of Black youth over a long period of time. FACHS, 1997–2017, followed 889 Black children aged 10–12 years old for up to 18 years. Depressive symptoms were measured in seven waves. The main predictors of interest were two SES indicators, parent education and family income measured at baseline (1997). Main outcome of interest was average depressive symptoms over the 18 year follow up period. Place of residence and gender were the focal moderators. Linear regression models were used for data analysis. In the pooled sample, living in a predominantly White area was associated with higher average depressive symptoms over time, however, this association was fully explained by higher perceived racial discrimination in the predominantly White areas. We found an interaction between income and place of residence on average depressive symptoms, suggesting that higher income is associated with more depressive symptoms in predominantly White compared to predominantly Black areas. Place did not interact with parent education on average depressive symptoms. Gender also did not interact with education or income on depressive symptoms. Findings suggest that place and SES may interact on depressive symptoms of Black youth, with high income becoming a risk factor for depressive symptoms in predominantly White areas. How SES indicators, such as income, protect or become a risk factor depend on other contextual factors, such as place of residence. There is a need to reduce discrimination experienced by Blacks, especially in predominantly White areas. Meanwhile, Black youth who live in predominantly White areas may require additional help that enhances their coping.

## 1. Background

The mainstream literature has traditionally conceptualized socioeconomic status (SES) indicators as protective factors [1]. For example, fundamental cause theory by Link and Phelan emphasizes the importance of high SES for maintaining population health [1]. With the same view, Mirowsky and Ross describe evidence behind protective health effects of SES as enduring, consistent, and growing [2]. Marmot and WHO’s Commission on Social Determinants of Health have emphasized social gradient in health, suggesting that health increases as SES increases, and health declines as SES declines [3,4,5]. These views are supported by several landmark studies in Europe [6,7,8,9] and the United States [7,10,11,12,13].

This is in part because most research on SES has traditionally focused on the overall effects of SES [14,15,16], leaving the heterogeneities in these effects still unknown. Some evidence suggests that sub-populations differ in their capacities to gain health from their SES resources [17,18]. Thus, there is a need to shift the attention from overall effects of SES [1,19,20,21] to sub-population variation in these effects [17,18,22]. While some research has documented different effects of SES in different populations [23], additional research is needed on whether the health effect of SES is conditional on race, ethnicity, gender, and place.

According to multiple disadvantage [24], cumulative disadvantage [25], double jeopardy [26], triple jeopardy [27], and multiple jeopardy [28] theories, SES is expected to generate more health gain for the racial and ethnic minority populations such as Blacks, as they are at a vulnerability status. These frameworks are suggestive of stronger effects of low SES among Blacks and other ethnic minority groups [28]. Contrasting these theories, however, the minorities’ diminished return theory expects smaller health effects of SES indicators for minority populations such as Blacks compared to Whites [17,18]. Not only may SES fail to protect mental health of Blacks [29,30,31,32,33,34], high SES may be a risk factor for depression, depressive symptoms, and suicide for Blacks, especially Black males [3,23,35,36]. For instance, in the National Survey of American Life (NSAL)–Adults sample, a positive association was found between family income and odds of major depressive disorder (MDD) [37]. In the NSAL–Adolescents sample, high family income was associated with higher MDD risk among Black boys [23].

Several mechanisms have been proposed for these unexpected observations of a positive link between SES and psychopathology of Blacks, particularly Black males. The first explanation is structural racism [38,39,40], which causes differential treatment of Blacks by society. For instance, Blacks are disproportionately discriminated against by the education system, housing market, labor market, and police [17]. A second explanation is that social, psychological, and physiological costs of upward social mobility are higher for Blacks compared to Whites [41]. This view is supported by a few studies that have documented weaker links between education attainment and health in Black compared to White youth [41,42]. There are also studies that show Blacks may use high-effort coping mechanisms for upward social mobility [43]. A third mechanism is interpersonal discrimination [44,45], i.e., perceived racial discrimination (PRD) that is higher at higher SES levels reduces the health gains that are typically produced by high SES [46]. Regardless of the exact mechanism, research has shown that SES indicators (e.g., education and income) may not only fail to generate health gain for Blacks [17,18], but may actually increase their risk for psychopathology [37]. Similarly, living in a safer neighborhood also shows a diminished gain for Blacks [47].

Place is a potential mechanism that may explain the positive link between SES and depression for Blacks [48]. In a recent study, Black youth who lived in predominantly White areas reported more PRD than those who lived in predominantly Black areas [48]. Place is shown to be linked to PRD [49,50,51,52], sleep [53], distress [54,55], stress [56], suicide [57], and depression [58]. SES aspects of the neighborhood, racial composition, physical and social environment, and segregation are all neighborhood and contextual factors that have mental health implications [59,60,61,62,63,64,65,66,67,68,69,70,71,72,73,74,75,76,77,78]. Ethnic density is one of the major factors that shapes PRD and depression for minorities including Blacks [49,54,79,80,81,82,83,84,85,86,87].

## 2. Aim

To replicate and extend previous empirical and theoretical work [17,18] and to better understand the non-linearity of the effects of race, gender, class, and place on mental well-being of Blacks in the U.S., this study examined the multiplicative effects of gender, place, and SES on subsequent burden of depressive symptoms over an extended period of time among Black youth. Using data from the used the Family and Community Health Study (FACHS), this study had two aims. Aim one was to determine whether living in a predominantly White area would be associated with more depressive symptoms, net of SES, and whether this effect could be explained by higher PRD in predominantly White areas. Aim two was to examine the interactions between place of residence and gender with SES on symptoms of depression. In this view, we conceptualized high SES, predominantly White neighborhoods, and male gender as vulnerability factors for risk of depression among Black youth.

## 3. Methods

### 3.1. Design and Setting

FACHS is an ongoing flagship longitudinal study of Black families in the U.S. The study recruited half of its sample from Iowa and half from Georgia, with an overall sample of 889 families at Wave 1 (year 1997) [88].

### 3.2. Ethical Considerations

The FACHS study protocol was approved by the Institutional Review Board (IRBs) at Iowa State where FACHS started. The study protocol was also approved by the IRB at University of Iowa, University of Georgia, and the University of Connecticut. Written assent was received from all participants through age 18 (Wave 4). Informed consent was received from youth above age 18 and also their parents or caregivers who participated in the study. Participants received financial compensation for their time and participation. 

### 3.3. Participants

Families who met inclusion criteria for this study were those with an adolescent in fifth grade at Wave 1 who self-identified as Black/African American and a primary caregiver willing to participate. Average age of the children at the time of enrollment in the study was 10.5 years. In 84% of the families, the parent was the biological mother of the adolescent. 

### 3.4. Recruitment

Participants were recruited to FACHS from rural communities, suburbs, and small metropolitan areas in Iowa and Georgia, composed of lower and middle class families. Median annual family income at the time of enrollment was $20,803/year, and one-third of families were below the poverty line. More detailed information regarding the FACHS sample and recruitment is available elsewhere [89,90].

### 3.5. Data Collection

Of the total families that were contacted, 72% provided data. The most common reason given for declining was the amount of time to complete interviews. All interviews were administered by Black/African American interviewers who had received extensive training. Length of interview, on average, was three hours. Data collection required one or two visits for interviews. The mode of interview was computer-assisted personal interview (CAPI), which increases quality of data when the questionnaire structure is complex [91]. Data were collected in Wave 1 (1997–1998), Wave 2 (1999–2000), Wave 3 (2002–2003), Wave 4 (2005–2006), Wave 5 (2007–2009), Wave 6 (2010–2011), and Wave 7 (2014–2016). Retention was 79% and 61% across the six and seven Waves. 

### 3.6. Measures

Sociodemographic characteristics—The following sociodemographic variables were included: age at Wave 1 (treated as a continuous measure with a range from 9 to 13), gender (male versus female [referent category]), and parent SES (annual family income and parent education), and place (Iowa versus Georgia [referent category]). Family income and education (highest education attainment of parents) were both treated as continuous measures. Regarding place, Georgia is 31% Black whereas Iowa is only 3.5% Black. (The neighborhoods in Iowa where FACHS recruited—urban areas of Des Moines and Waterloo—had higher % Black than the rest of the state, which is <1% Black).

Perceived Racial Discrimination (PRD)—Participants reported their experience of racist events, using the 13-item modified version of the Schedule of Racist Events (SRE) scale [92]. After describing various discriminatory events, participants were asked how often they have experienced each of the events. Example item includes “How often has someone said something insulting to you just because you are African American?” Item responses ranged from 1 (never) to 4 (several times) (αs = 0.86–0.90). PRD measured using this scale is shown to predict poor health [88,93,94].

Depressive symptoms—Youth’s depressive symptoms were measured up to Wave 4 using the Diagnostic Interview Schedule for Children, Version IV (DISC-IV) [95]. The DISC-IV has shown acceptable reliability and validity for measurement of depression [95]. During an interview, youth were asked 20 questions about their mood and affect during the past year. All items used a three-point response scale (0 = no, 1 = sometimes, 2 = yes) [96]. We created a sum score with higher score indicating more depressive symptom counts. As number of items were not identical across waves, we calculated z scores. Our outcome was average depressive symptoms over all seven waves. Starting at Wave 5, a brief version of the UM-CIDI was used to measure depressive symptoms. Higher score was indicative of more chronic depression over time. 

### 3.7. Statistical Analysis

Data analysis was conducted using SPSS 22.0 (SPSS, Chicago, IL, USA). We used frequency tables and descriptive characteristics to describe our sample. We used Pearson correlation test for bivariate associations. We used eight linear regression models for our multivariable analysis. Adjusted unstandardized regression coefficients (B), 95% confidence interval (CI), and *p* values were reported. *p* value of less than 0.05 was considered statistically significant. In our regression models, average depressive symptoms over the 18 year follow up period was the dependent variable. The main independent variables of interest were two SES indicators namely parent education and family income at baseline. Place and gender were focal moderators. To test associations of interest, first we ran Model 1 to Model 4 in the pooled sample. Then we ran Model 5 to Model 8 by place. First, our models tested the main effects of SES indicators in the absence of interactions. Then, the models tested the interactive effects of place, gender, and SES.

## 4. Results

### 4.1. Descriptive Statistics

Table 1 summarizes descriptive statistics for demographics, SES, PRD, baseline depressive symptoms, and average depressive symptoms over time in the pooled sample (Table 1).

### 4.2. Bivariate Correlations

Table 2 shows the results of bivariate correlations between all study measures in the pooled sample, as well as based on place of residence. Residence in Iowa (predominantly White areas) was associated with higher parent education but not family income. Residence in Iowa was also associated with higher average PRD over the follow up period and higher depressive symptoms at baseline and throughout the follow up period. In Georgia, higher family income and parent education were correlated with lower average depressive symptoms over time. Such correlations were not found in Iowa (Table 2).

### 4.3. Linear Regressions (Main Effect Models) in the Pooled Sample

Table 3 shows the results of two linear regression models (Model 1 and Model 2) in the pooled sample to test the additive effects of place of residence, SES, gender, and PRD on average depressive symptoms over the follow up period. These models only included main effects. Model 1 did not include average PRD in the model, whereas, Model 2 did include average PRD in the model (Table 3).

In Model 1, living in Iowa (predominantly White areas) was associated with higher average depressive symptoms over the follow up period, independent of other covariates. In Model 2, the association between living in Iowa and average depression over the follow up period was fully explained by average PRD. This suggests that average PRD fully mediates the effect of living in Iowa (predominantly White areas) on average depressive symptoms over the follow up period (Table 3).

### 4.4. Linear Regressions (Interaction Models) in the Pooled Sample

Table 4 shows the results of two linear regression models (Model 3 and Model 4) in the pooled sample to test the multiplicative effects of place of residence, SES, and PRD on average depressive symptoms over the follow up period. These models included main effects as well as two by two interaction terms among gender, place, and SES indicators (parent education and family income). Model 3 did not include average PRD, while Model 4 did (Table 4).

Model 3 and Model 4 showed a significant and positive interaction between living in Iowa and higher family income on average depressive symptoms over the follow up period, suggesting that higher income is associated with more depressive symptoms in Iowa, compared to Georgia. As the models with and without PRD did not differ, the interaction between place and income could not be attributed to PRD (Table 4).

### 4.5. Linear Regressions (Without PRD) in Georgia and Iowa

Table 5 shows the results of two linear regression models in Georgia (Model 5) and Iowa (Model 6). These models included two by two interaction terms between gender and SES (parent education and family income). These models, however, did not include PRD. Family income was associated with lower average depressive symptoms over the follow up period in Georgia (Model 5) but not in Iowa (Model 6) (Table 5).

Table 6 shows the results of two linear regression models in in Georgia (Model 7) and Iowa (Model 8). These models included two by two interaction terms between gender and SES (education and income) as well as PRD. When PRD was controlled, high income was associated with lower depressive symptoms over the follow up period in Georgia (Model 7) but not Iowa (Model 8) (Table 6).

## 5. Discussion

Our study revealed three major findings: First, residence in predominantly White areas was associated with more average depressive symptoms over a long follow up period, an effect which was fully explained by higher PRD reported by Black youth in predominantly White areas. Second, an interaction was found between place of residence and SES (income) on depressive symptoms, suggesting that high income may become a risk factor for experiencing higher depressive symptoms, on average, over a long follow up period for Black youth who live in predominantly White areas. This finding was also replicated in stratified models based on place. For example, in Iowa, family income showed an inverse association with average depressive symptoms, while in Georgia income was negatively associated with average depressive symptoms over time. Third, parent education was protective against depressive symptoms in Iowa but not Georgia. 

The higher average depressive symptoms of Black youth from high income families and those who live in predominantly White areas is not supported by the mainstream literature on protective effects of SES [1,2,3]. Our finding invites theories such as FCT to consider sub-population variability in the SES—health link [1]. Our finding does, however, support recent literature documenting higher risk of depression, depressive symptoms, and suicide in high SES Blacks, particularly Black males [23,33,34,37]. In a national survey, high income was protective against risk of MDD for Whites but not Blacks [97]. In a study of Michigan residents, high income was protective against poor self-rated mental health for Whites but not Blacks [33]. 

These findings indirectly suggest that density of Whites in the area may be a risk factor for higher discrimination and depression among Black youth over time. In a study using the FACHS data, Black youth from high SES and those who were living in predominantly White areas reported more discrimination than others [48]. This hypothesis is supported by the studies that have explored residential preferences of Blacks and Whites in the US. In a study by Maria Krysan and Reynolds Farley, Blacks’ preference to live in predominantly Black neighborhoods was mainly driven by fears of White hostility rather than preference due to solidarity or neutral ethnocentrism [30]. Future research should directly test the role of % Black and White as determinant of discrimination and mental health of Black youth [98]. Such studies can also contribute to the ongoing debate between same-ethnicity preferences versus discrimination as a main cause of residential segregation [99,100,101,102,103]. 

High SES should not be assumed as a universal protective factor across all outcomes, populations, and settings. Population sociodemographic characteristics such as race do not add but interact with the effects of SES indicators, such as education and income, on population health [104,105]. Rather than control variables, SES and place should be considered contextual factors that reflect populations’ exposure, susceptibility, vulnerability, and resilience [17,18]. In statistical terms, race, SES, and place should be conceptualized as effect modifiers (moderators) that change the direction or magnitude of the effects of risk and protective factors [106]. These findings advocate for application of the intersectionality framework to study experiences and vulnerabilities across each unique sub-section of the society [107,108].

Recent research has documented poor mental health of high SES Blacks, particularly men [35,109,110]. In the NSAL–Adolescents sample, a positive association was found between family income and MDD risk among Black boys [23]. In the NSAL–Adults sample, income was positively linked to MDD risk in Black men [23]. In another study using the NSAL–Adults sample, high education was positively associated with suicidality in Black women [35]. In the Health Retirement Survey (HRS), two SES indicators (education and income) showed protective effects on five health outcomes more consistently for White men and women, compared to the weak or absent protective effects for Black men and women. In that study, income did not show any protective effects for Black men against high BMI. High education attainment was protective for sleep, physical activity, and BMI for all race by gender groups with Black men being the only exception [109]. In the Religion and Aging Health Study (RAHS), high education showed an effect on drinking behaviors of Whites, but not Blacks [106]. In the Americans Changing Lives (ACL) study, among Black men, high education was associated with an increase in subsequent depressive symptoms over time [110]. In the ACL, education [111], employment [112], neighborhood [47], and social contacts [113] better reduced mortality for Whites than Blacks. Other studies have also documented racial differences in the effects of education on mortality [114]. In the Fragile Families and Child Wellbeing Study (FFCWS), family structure and maternal education improved BMI [115], SRH [31], and impulse control [30] for White but not Black families. In the FFCWS, White mothers were better able to use education, marital status, and health insurance, to escape hunger and breastfeed their newborn child compared to Black mothers [116]. In another national study, education helped White parents to escape poverty more than it did Black parents [117]. These are in part because education generates less income for Blacks than Whites [118] and highly educated Blacks are more likely to stay poor than highly educated Whites [119]. 

Several mechanisms could explain findings of poorer mental health of high income Blacks [17,18]. The first possible explanation is the extra social, psychological, and social costs of upward social mobility for Black individuals. Research by Fuller Rowel and colleagues has suggested that population health does not improve as significantly in Blacks, compared to Whites, as their SES increases [41,42]. A second explanation is the tendency of Blacks to use high-effort coping strategies, such as John Henryism [43,120]. The Black population also has a higher tendency to goal-striving stress, which comes with psychological costs [121]. Additional research is needed on how race, gender, class, and place shape coping behaviors in minorities. The third explanation is additional exposure and vulnerability to PRD among high SES Blacks. Hudson et al. have shown that Blacks report more, not less, PRD as their SES improves [46]. The same study has shown that high PRD reduces the health gain from SES [46]. This is particularly relevant to Black men, as male gender is also shown to be a vulnerability factor to PRD [107,122,123,124].

Society treats Blacks differently than Whites. Compared to Whites, Blacks have less access to the opportunity structure. The change of life circumstances due to upward social mobility is not equal across races. Such effects largely depend on the availability of other SES resources, which drastically differ between Whites and Blacks [111]. SES resources, such as education and income, have smaller effects on changing life conditions, life styles, and behaviors, generating wealth, and increasing purchasing power for Blacks than Whites [35,106,110,111,125]. This is another reason SES indicators have shown smaller protective effects on drinking [106], depressive symptoms [110], suicidality [35], chronic disease [110], and mortality [126] for Blacks than Whites.

We found some protective effects of SES indicators on depressive symptoms in the pooled sample and in Black youth in Georgia. We also found protective effects of parental education against chronic depressive symptoms in Iowa (Model 6 and Model 8), a finding supported by extensive theoretical and empirical work. FCT [1,19], for example, considers SES as a fundamental cause of health [19,20,21]. Many state-of-the-art longitudinal studies have shown that SES indicators, such as education and income, protect populations against a wide range of undesired health outcomes [6,7,8,9] including but not limited to depression [127].

There are multiple mechanisms explaining protective effects of SES on mental health. High SES means lower exposure to a wide range of environmental risk factors [128,129,130]. High SES individuals have access to resources that can be used to avoid risk factors and harmful exposures [1,19], including general and financial stress [131]. In addition to reducing exposure to stress, SES buffers the effects of exposures when they are faced [20,21,132,133]. Higher SES reflects higher human capital [132], healthy behaviors [134], and a larger social network [42]. SES resources also enhance access to healthcare when needed [135]. Many of these effects of SES on health [1,19,20,21], however depend on race [17,18]. For Blacks, SES is positively associated with PRD, which itself is associated with unhealthy behaviors [136,137,138,139], poor mental [140,141,142,143] and physical health [144].

## 6. Limitations

Although the current findings extend the existing literature on the intersection of race, class, and place, the results should be interpreted in the light of the study limitations. The first limitation was that place was operationalized as a dichotomous variable in this study, which is over-simplistic. In our study, place was not an ideal a proxy of density of Black people (vs. density of White population). There is a need to study how racial composition, social, and physical aspects of neighborhood and other contextual factors contribute to the risk of PRD and depression for Black youth. As the variable place in our study consisted of two American States only (Iowa vs. Georgia), we still cannot attribute its effects to the percentage of White and Black people in the two States. More research is needed to confirm this hypothesis. Future research should explore the gradations and also the thresholds of the effects of neighborhood characteristics in shaping Black youth exposure to PRD. Future research should also examine higher level SES indicators such as area level poverty and unemployment. In addition to racial composition of the neighborhood, other aspects of social environment—such as cohesion, socialization, trust, and coping—merit further evaluation. Another limitation was conceptualization of the dependent variable as average depressive symptoms over a long period of time. As conceptualized in other domains and also depression [79,109,145,146], average depressive symptoms over 18 years (7 waves) could reflect chronic depression over time. However, participants with the same average could be of several types: high scores at the early waves; high scores at the later waves; consistent scores across waves—each could have different implications. As a result, future research should explore heterogeneity by modeling the latent classes of depressive symptoms over time. Finally, validity of measures of depressive symptoms are a function of class, so the positive association between income and depressive symptoms may be confounded by lower social desirability and higher mental health literacy of high SES Black youth. Despite these limitations, our analysis is one of the few that uses a long term longitudinal study to explore the multiplicative effects of place, gender, and SES on depression.

## 7. Conclusions

In summary, we found evidence suggesting that place and SES interact on depressive symptoms, and high SES (family income) may operate as a risk factor for depressive symptoms among Black youth who live in predominantly White areas. It is, however, unknown how and why high income increases depressive symptoms of Black families who live in such areas. Future research should explore the particular contextual factors that make high SES a vulnerability factor for Black youth.

## Figures and Tables

**Table 1:** Descriptive statistics in the pooled sample (*n* = 889).

	*N*	Minimum-Maximum	Mean (SD)
Age (Years)	837	9.00–13.00	10.36 (0.71)
Parent Education ^a^ (1–7)	806	1.00–7.00	3.23 (1.13)
Annual Family Income ^a^ (1000 USD)	784	0.00–221.10	28.47 (25.61)
Mean *z* score Discrimination	884	−1.19–3.68	0.00 (0.73)
Baseline *z* score Depression	878	−1.27–3.13	0.00 (1.00)
Mean *z* score Depression	888	−1.35–2.71	0.00 (0.66)

Source: Family and Community Health Study (FACHS); ^a^ Reported by the primary caregiver, almost always mother of the participating youth.

**Table 2:** Correlation matrix of the study variables (*n* = 889).

	1	2	3	4	5	6	7	8
All (*n* = 889)								
1—Place of Residence (Iowa)	1.00	0.08 *	0.00	0.18 **	0.03	0.31 **	0.19 **	0.15 **
2—Age (Years)		1.00	−0.01	0.03	0.03	0.06 ^#^	0.00	0.00
3—Gender (Female)			1.00	0.02	−0.01	0.04	−0.01	−0.18 **
4—Parent Education (1–7) ^a^				1.00	0.43 **	0.01	0.02	−0.06 ^#^
5—Family Income (USD) ^a^					1.00	−0.05	−0.05	−0.08 *
6—Average Perceived Racial Discrimination (Wave 1–7)						1.00	0.27 **	0.39 **
7—Baseline Depression (Wave 1)							1.00	0.56 **
8—Average Depression (Wave 1–7)								1.00
Georgia								
2—Age (Years)		1.00	0.04	0.04	−0.05	0.03	0.00	0.00
3—Gender (Female)			1.00	0.05	−0.03	0.10 *	−0.07	−0.15 **
4—Parent Education (1–7) ^a^				1.00	0.43 **	0.03	−0.04	−0.12 *
5—Family Income (USD) ^a^					1.00	−0.06	−0.08	−0.15 **
6—Average Perceived Racial Discrimination (Wave 1–7)						1.00	0.23 **	0.40 **
7—Baseline Depression (Wave 1)							1.00	0.51 **
8—Average Depression (Wave 1–7)								1.00
Iowa								
2—Age (Years)		1.00	−0.06	−0.01	0.09 ^#^	0.04	-0.03	−0.03
3—Gender (Female)			1.00	−0.01	0.00	0.00	0.04	−0.21 **
4—Parent Education (1–7) ^a^				1.00	0.43 **	−0.11 *	0.01	−0.06
5—Family Income (USD) ^a^					1.00	−0.06	−0.03	−0.03
6—Average Perceived Racial Discrimination (Wave 1–7)						1.00	0.22 **	0.36 **
7—Baseline Depression (Wave 1)							1.00	0.57 **
8—Average Depression (Wave 1–7)								1.00

Source: Family and Community Health Study (FACHS); ^a^ Reported by the primary caregiver, almost always mother of the participating youth. ^#^ *p* < 0.1; * *p* < 0.05; ** *p* < 0.01.

**Table 3:** Summary of linear regressions to test the main effects of place, socioeconomic status (SES), gender, and discrimination on average depressive symptoms over 18 years (*n* = 889).

	Model 1			Model 2		
	b	95% CI	*p*	b	95% CI	*p*
Gender (Male)	−0.26	−0.34–0.18	<0.001	−0.27	−0.35–0.20	<0.001
Age (Year)	0.00	−0.05–0.06	0.861	−0.01	−0.07–0.04	0.610
Place of Residence (Iowa)	0.08	0.00–0.17	0.048	−0.02	−0.10–0.06	0.664
Family Income (1000 USD) ^a^	−0.42	−2.12–1.28	0.629	−0.19	−1.81–1.43	0.815
Parent Education ^a^	−0.05	−0.09–0.01	0.013	−0.05	−0.08–0.01	0.018
Depression (*z* score, W1)	0.34	0.30–0.39	<0.001	0.31	0.27–0.35	<0.001
Discrimination (*z* score, W1–W7)	-	-	-	0.25	0.20–0.31	<0.001
Intercept	0.21	−0.38–0.80	0.482	0.44	−0.12–1.00	0.124

Source: Family and Community Health Study (FACHS); b: Unstandardized B coefficient; ^a^ Reported by the primary caregiver, almost always mother of the participating youth.

**Table 4:** Summary of linear regressions to test the interactive effects of place, socioeconomic status (SES), gender, and discrimination on average depressive symptoms over 18 years (*n* = 889).

	Model 3			Model 4		
	B	95% CI	*p*	B	95% CI	*p*
Gender (Male)	−0.26	−0.51–0.01	0.040	−0.25	−0.48–0.02	0.036
Age (Year)	0.00	−0.06–0.06	0.993	−0.02	−0.07–0.04	0.507
Place of Residence (Iowa)	0.04	−0.21–0.29	0.770	−0.17	−0.41–0.08	0.178
Family Income (1000 USD) ^a^	−1.66	−4.33–1.01	0.222	−1.09	−3.63–1.44	0.398
Parent Education ^a^	−0.05	−0.11–0.02	0.169	−0.06	−0.12–0.01	0.078
Family Income (1000 USD) × Male	−1.41	−4.95–2.13	0.435	−1.66	−5.02–1.70	0.333
Family Income (1000 USD) × Place	3.83	0.28–7.37	0.035	3.37	0.00–6.74	0.050
Parent Education × Male	0.01	−0.07–0.09	0.784	0.01	−0.07–0.08	0.845
Parent Education × Place	−0.02	−0.10–0.06	0.644	0.02	−0.06–0.09	0.683
Depression (*z* score, W1)	0.34	0.30–0.38	<0.001	0.31	0.27–0.34	<0.001
Discrimination (*z* score, W1–W7)	-	-	-	0.26	0.20–0.31	<0.001
Intercept	0.29	−0.33–0.90	0.357	0.54	−0.04–1.13	0.069

Source: Family and Community Health Study (FACHS); B: Unstandardized B coefficient; ^a^ Reported by the primary caregiver, almost always mother of the participating youth.

**Table 5:** Summary of linear regressions on the interactive effects of SES and gender on average depressive symptoms over 18 years by place (*n* = 889).

	Georgia			Iowa		
	Model 5			Model 6		
	B	95% CI	*p*	B	95% CI	*p*
Gender (Male)	−0.42	−0.75–0.09	0.013	−0.21	−0.59–0.16	0.270
Age (Year)	0.04	−0.05–0.12	0.408	−0.03	−0.10–0.04	0.424
Family Income (1000 USD)	−0.09	−0.17–0.01	0.022	−0.06	−0.14–0.03	0.168
Parent Education	−2.11	−5.03–0.82	0.157	3.88	0.07–7.69	0.046
Family Income (1000 USD) × Male	0.64	−4.43–5.71	0.804	−3.88	−8.79–1.02	0.120
Parent Education × Male	0.08	−0.03–0.19	0.165	−0.01	−0.13–0.10	0.821
Depression Baseline	0.31	0.26–0.37	<0.001	0.38	0.32–0.43	<0.001
Intercept	0.00	−0.90–0.90	0.996	0.61	−0.22–1.44	0.150

Source: Family and Community Health Study (FACHS); B: Unstandardized B coefficient; ^a^ Reported by the primary caregiver, almost always mother of the participating youth.

**Table 6:** Summary of linear regressions on the interactive effects of SES, gender, and discrimination on average depressive symptoms over 18 years by place (*n* = 889).

	Georgia			Iowa		
	Model 7			Model 8		
	B	95% CI	*p*	B	95% CI	*p*
Gender (Male)	−0.38	−0.68–0.08	0.014	−0.20	−0.57–0.16	0.275
Age (Year)	0.00	−0.08–0.08	0.981	−0.04	−0.11–0.03	0.291
Family Income (1000 USD) ^a^	−0.09	−0.16–0.02	0.014	−0.05	−0.13–0.04	0.279
Parent Education ^a^	−1.73	−4.41–0.96	0.207	4.19	0.50–7.88	0.026
Family Income (1000 USD) × Male	1.38	−3.28–6.03	0.561	−4.55	−9.32–0.22	0.062
Parent Education × Male	0.04	−0.06–0.15	0.407	−0.01	−0.12–0.10	0.901
Depression Baseline	0.27	0.21–0.32	<0.001	0.34	0.29–0.40	<0.001
Discrimination (*z* score, W1–W7)	0.37	0.28–0.47	<0.001	0.19	0.12–0.26	<0.001
Intercept	0.45	−0.39–1.28	0.293	0.60	−0.21–1.41	0.145

Source: Family and Community Health Study (FACHS); B: Unstandardized B coefficient; ^a^ Reported by the primary caregiver, almost always mother of the participating youth.
